# Domestic

**Published:** 1841-01-23

**Authors:** 


### DOMESTIC.

*Introductory Lectures.*—We acknowledge the receipt of introductory lectures by Dr. James McClintock, of Philadelphia, and Professor Frank H. Hamilton, of Geneva Medical College, New York. Dr. McClintock is one of the private teachers of this city, who, unconnected with any of the chartered schools, are laboriously and usefully employed in completing the education of some of the multitude of students who resort hither. The lecture before us, introductory to Dr. McClintock's winter course of anatomy, is an appropriate and spirited appeal to the student on the value of anatomical knowledge. To the physician the topic is trite, and we forbear further notice.

We have read with pleasure previous efforts of *Professor Hamilton*'s, and are happy to bear testimony to the merit of the introductory before us, which contains much wholesome advice on the moral and physical training of the young surgeon.

*Harvard University.*—The annual circular for 1840-41, represents the medical department of this institution in a very flourishing condition—eighty-eight students having matriculated at the present session.

---

*Pepperell Institution for the Insane.*—In the year 1825, Dr. Cutler, of Pepperell, Mass., a town 40 miles N. W. of Boston, without particularly wishing to embrace within the circle of his practice the diseases of the mind, unexpectedly found himself so constantly consulted by the friends of the insane, in consequence of the fortunate manner in which he had treated a few marked cases, that he was actually obliged to make some extra provision for the accommodation of that class of patients. We had contemplated a minute historical account of this now quite celebrated asylum, but it would occupy too much room in the Journal, without being essential to the object at first contemplated in this notice.

The rules and regulations which were instituted at the commencement for the government of the Asylum, are appended, to show the complete organization of the establishment, which has grown extensively into public favour, and continues to maintain the character that we trust it will always sustain.

"1st. It is the duty of the attendants to devote their whole time to the boarding patients. Let your conduct to the patients be always civil, respectful and polite. Use no unnecessary authority. Let your language be calm and persuasive. Endeavor to control them by persuasion; and if compulsion be necessary, use it with great care and wisdom, remembering to explain to them the reason and intention of your procedure. It is your duty to exercise a moral influence over the patients, and endeavour to improve and amuse their minds. When they are in their rooms, amuse them by reading some appropriate book, by chess, checkers, shuttlecock, ninepins, &c. Strict attention to be paid to their dress, &c. Particular attention given to them when they ride or walk. Attend to their deportment, and correct all improper talk and conduct. Never call at stores or any public house, and have no conversation with the people on the way. Never appear pleased with any indecent language or behaviour, but always show your disapprobation.

"2d. All the boarders, in a suitable state of mind, must attend morning and evening devotion, at the ringing of the bell for prayers, accompanied by their nurses and attendants—and also attend church on Sundays.

"3d. *Exercises.*—Their exercise must commence immediately after morning devotion. Those whol abour on the farm and in the workshop, go to their business under the direction of their attendants; and those who exercise by riding, walking, &c., with their attendants. All must return to their rooms at 11, A. M., to be in readiness for dinner at 12, M. All the patients in a suitable state of mind, dine at the family table under the direction of the physician, Dr. C., and those who are not, in their rooms under the direction of their attendants.

"4th. *Afternoon exercises* commence at two o'clock, and are somewhat similar to those of the forenoon, (in the summer season,) with the addition of amusements in the grove, such as bowling, swinging, and riding the flying horses. All retire to their rooms at five o'clock, and prepare for supper, which they take in the same manner as dinner and breakfast.

"5th. At half past eight o'clock all attend evening devotion, and at nine retire to their beds."

With regard to the internal conveniences and medical treatment, a few items are subjoined, amply sufficient to show that the comfort and well-being of those confided to Dr. Cutler's care are promptly attended to.

"The course of treatment from the commencement has been adapted to the derangement of the physical system, and a strict attention paid to the moral management of the patients. One principle has been to treat them as sane, and as ladies and gentlemen. Their exercise, which always has been considered an important agent in the curative process, is walking, riding on horseback and in carriages, bowling, swinging, &c., and also manual labour on the farm, which is one of the best exercises in use. The workshop has also been in use."

About five hundred and twenty-eight patients have been received since 1825; and not far from nine-tenths, it is supposed, have been discharged well, or greatly improved.

In the year 1839, Dr. Parker, a pupil of Dr. Cutler's, became a joint partner in the concerns of the institution, which still continues, under their management, one of the best private hospitals for the insane in the country; and it shows how much can be accomplished by perseverance in the cause of humanity, on the part of a single individual.—*Bost. Med. and Surg. Journ.*

